# 2-[(*E*)-({4-[(4,6-Dimethyl­pyrimidin-2-yl)sulfamo­yl]phen­yl}iminio)meth­yl]-6-hy­droxy­phenolate

**DOI:** 10.1107/S1600536812034757

**Published:** 2012-08-11

**Authors:** M. Nawaz Tahir, Abdul Haleem Khan, Mohammad S. Iqbal, Hazoor Ahmad Shad, Muhammad Yaqub

**Affiliations:** a Department of Physics, University of Sargodha, Sargodha, Pakistan; b Department of Pharmacy Services, Jinnah Hospital, Lahore, Pakistan; c Department of Chemistry, Forman Christian College, Lahore 54600, Pakistan; d Department of Chemistry, Government Post Graduate College, Gojra, Punjab, Pakistan; e Department of Chemistry, Bahauddin Zakariya University, Multan 60800, Pakistan

## Abstract

The title compound, C_19_H_18_N_4_O_4_S, exists as a zwitterion in the solid state, with nominal proton transfer from a phenol group to the imine N atom. The 2,3-dihy­droxy­benzaldehyde fragment is oriented at a dihedral angle of 35.51 (11)° to the adajacent aniline group and makes a dihedral angle of 76.99 (6)° with the 4,6-dimethyl­pyrimidin-2-amine group. Intra­molecular O—H⋯O and N—H⋯O hydrogen bonds close *S*(5) and *S*(6) rings, respectively; the same O atom accepts both bonds. In the crystal, polymeric chains along [001] are formed from mol­ecules joined end-to-end by N—H⋯O and O—H⋯N hydrogen bonds; these feature *R*_2_^3^(6) loops. The polymeric chains are linked by C—H⋯O inter­actions and there are π–π inter­actions between the pyrimidine rings with a centroid–centroid distance of 3.446 (2) Å.

## Related literature

For related structures, see: Chohan *et al.* (2008 ▶); Shad *et al.* (2009 ▶); Tahir *et al.* (2012 ▶). For graph-set notation, see: Bernstein *et al.* (1995 ▶).
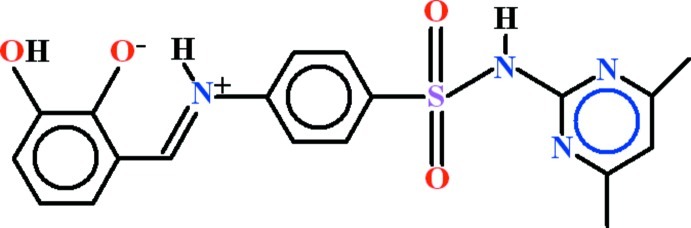


## Experimental

### 

#### Crystal data

C_19_H_18_N_4_O_4_S
*M* *_r_* = 398.43
Orthorhombic, 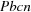

*a* = 24.7506 (12) Å
*b* = 12.1689 (6) Å
*c* = 12.8408 (5) Å
*V* = 3867.5 (3) Å^3^
*Z* = 8
Mo *K*α radiation
μ = 0.20 mm^−1^
*T* = 296 K
0.34 × 0.28 × 0.15 mm

#### Data collection

Bruker Kappa APEXII CCD diffractometer
Absorption correction: multi-scan (*SADABS*; Bruker, 2005 ▶) *T*_min_ = 0.935, *T*_max_ = 0.971
16627 measured reflections
3796 independent reflections
1778 reflections with *I* > 2σ(*I*)
*R*_int_ = 0.069

#### Refinement

*R*[*F* ^2^ > 2σ(*F* ^2^)] = 0.054
*wR*(*F* ^2^) = 0.151
*S* = 1.01
3796 reflections
259 parameters
H atoms treated by a mixture of independent and constrained refinement
Δρ_max_ = 0.21 e Å^−3^
Δρ_min_ = −0.28 e Å^−3^

### 

Data collection: *APEX2* (Bruker, 2007 ▶); cell refinement: *SAINT* (Bruker, 2007 ▶); data reduction: *SAINT*; program(s) used to solve structure: *SHELXS97* (Sheldrick, 2008 ▶); program(s) used to refine structure: *SHELXL97* (Sheldrick, 2008 ▶); molecular graphics: *ORTEP-3 for Windows* (Farrugia, 1997 ▶) and *PLATON* (Spek, 2009 ▶); software used to prepare material for publication: *WinGX* (Farrugia, 1999 ▶) and *PLATON*.

## Supplementary Material

Crystal structure: contains datablock(s) global, I. DOI: 10.1107/S1600536812034757/hb6908sup1.cif

Structure factors: contains datablock(s) I. DOI: 10.1107/S1600536812034757/hb6908Isup2.hkl

Supplementary material file. DOI: 10.1107/S1600536812034757/hb6908Isup3.cml

Additional supplementary materials:  crystallographic information; 3D view; checkCIF report

## Figures and Tables

**Table 1:** Hydrogen-bond geometry (Å, °)

*D*—H⋯*A*	*D*—H	H⋯*A*	*D*⋯*A*	*D*—H⋯*A*
N1—H1⋯O1	0.78 (4)	1.88 (4)	2.569 (5)	148 (4)
O2—H2⋯O1	0.82	2.34	2.768 (5)	113
O2—H2⋯N3^i^	0.82	2.16	2.862 (5)	144
N2—H2*A*⋯O1^ii^	0.86	1.94	2.790 (4)	172
C18—H18*A*⋯O4^iii^	0.96	2.52	3.469 (5)	171

Symmetry codes: (i) 
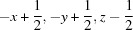
; (ii) 
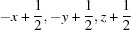
; (iii) 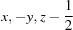.

**Fig. 1.**
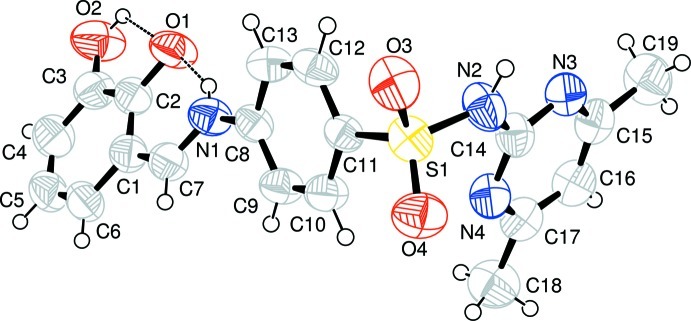
View of the title compound with displacement ellipsoids drawn at the 50% probability level. The dotted lines represent the intramolecular H-bonds.

**Fig. 2.**
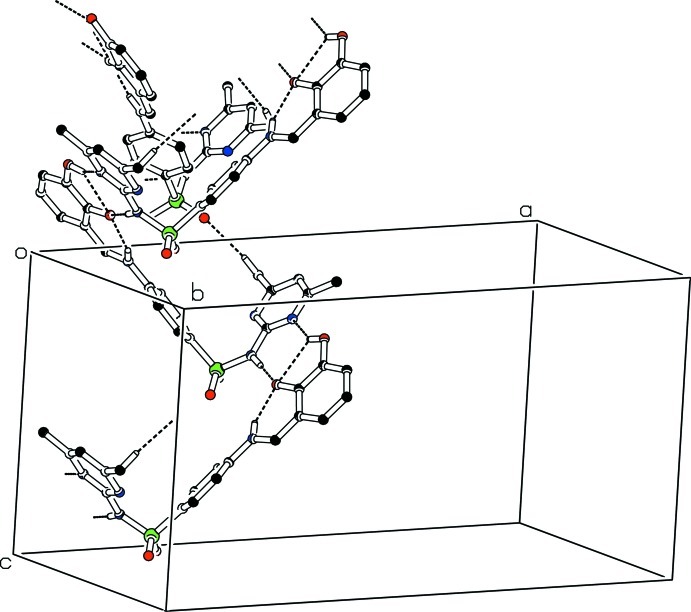
The partial packing, which shows that molecules form polymeric chains along [001]. The H-atoms not involved in H-bondings are omitted for clarity.

## supplementary crystallographic information

### Comment

We have reported the crystal structure of 4-{[(*E*)-(2,3-dihydroxyphenyl) methylidene]amino}-*N*-(5-methyl-1,2-oxazol-3-yl)benzenesulfonamide (Tahir *et al.*, 2012) and the title compound (I), (Fig. 1) has also been synthesized for the biological studies and forming different metal complexes.

The crystal structures of 4-(5-chloro-2-hydroxybenzylideneamino)-*N*- (4,6-dimethylpyrimidin-2-yl)benzenesulfonamide (Chohan *et al.*, 2008) and 4-[(5-bromo-2-hydroxybenzylidene)amino]-*N*-(4,6-dimethylpyrimidin- 2-yl)benzenesulfonamide–4-bromo-2-[(*E*)-({4-[(4,6-dimethylpyrimidin-2-yl) sulfamoyl]phenyl}iminio)methyl]phenolate (Shad *et al.*, 2009) have been published which are related to the title compound.

In (I) the parts of 2,3-dihydroxybenzaldehyde A (C1—C7/O1/O2), annilinic group B (C8—C13/N1) and 4,6-dimethylpyrimidin-2-amine C (C14—C19/N2/N3/N4) are planar with r.m.s. deviation of 0.0105, 0.0070 and 0.0216 Å, respectively. The dihedral angle between A/B, A/C and B/C is 35.51 (11)°, 76.99 (6)° and 88.92 (6)°, respectively. The sulfonyl group D (O3/S1/O4) is of course planar. The dihedral angle between A/D, B/D and C/D is 62.20 (13)°, 47.66 (17)° and 50.34 (15)°, respectively. In (I), *S*(5) and *S*(6) ring motif (Bernstein *et al.*, 1995) are present due to H-bondings of O—H···O and N—H···O types, respectively (Table 1, Fig. 1). The molecules are interlinked from end to end due to H-bondings of N—H···O and O—H···O types (Table 1, Fig. 2). Due to these bondings *R*_2_^3^(6) loops are also formed. The molecules are interlinked in the form of polymeric chains along the *c*-axis. The polymeric chains are also interlinked due to C–H···O bondings (Table 1, Fig. 2), Where CH is of methyl group and O-atom is of sulfonyl group. There exist π–π interaction between *Cg*1···*Cg*1^i^ [i = 1 - *x*, *y*, 1/2 - *z*] at a distance of 3.446 (2) Å, where *Cg*1 is the centroid of pyrimidin ring (C14—C17/N3/N4).

### Experimental

Equimolar quantities of 4-amino-*N*-(4,6-dimethylpyrimidin-2-yl) benzenesulfonamide (Sulfamethazine) and 2,3-dihydroxybenzaldehyde were refluxed in methanol along with few drops of acetic acid as catalyst for 3 h. The solution was kept at room temperature which afforded dark red plates after four days upon slow evaporation of the solvent.

### Refinement

The coordinates of H1 were refined. The H-atoms were positioned geometrically (C–H = 0.93–0.96 Å, N—H= 0.86 Å, O—H= 0.82 Å) and refined as riding with *U*_iso_(H) = *xU*_eq_(C, N, O), where *x* = 1.5 for hydroxy & methyl and *x* = 1.2 for other H-atoms.

### Crystal data

**Table:**

C_19_H_18_N_4_O_4_S	*F*(000) = 1664
*M**_r_* = 398.43	*D*_x_ = 1.369 Mg m^−^^3^
Orthorhombic, *P**b**c**n*	Mo *K*α radiation, λ = 0.71073 Å
Hall symbol: -P 2n 2ab	Cell parameters from 1778 reflections
*a* = 24.7506 (12) Å	θ = 1.7–26.0°
*b* = 12.1689 (6) Å	µ = 0.20 mm^−^^1^
*c* = 12.8408 (5) Å	*T* = 296 K
*V* = 3867.5 (3) Å^3^	Plate, dark red
*Z* = 8	0.34 × 0.28 × 0.15 mm

### Data collection

**Table:**

Bruker Kappa APEXII CCD diffractometer	3796 independent reflections
Radiation source: fine-focus sealed tube	1778 reflections with *I* > 2σ(*I*)
Graphite monochromator	*R*_int_ = 0.069
Detector resolution: 8.00 pixels mm^-1^	θ_max_ = 26.0°, θ_min_ = 1.7°
ω scans	*h* = −30→29
Absorption correction: multi-scan (*SADABS*; Bruker, 2005)	*k* = −15→15
*T*_min_ = 0.935, *T*_max_ = 0.971	*l* = −15→14
16627 measured reflections	

### Refinement

**Table:**

Refinement on *F*^2^	Primary atom site location: structure-invariant direct methods
Least-squares matrix: full	Secondary atom site location: difference Fourier map
*R*[*F*^2^ > 2σ(*F*^2^)] = 0.054	Hydrogen site location: inferred from neighbouring sites
*wR*(*F*^2^) = 0.151	H atoms treated by a mixture of independent and constrained refinement
*S* = 1.01	*w* = 1/[σ^2^(*F*_o_^2^) + (0.0534*P*)^2^ + 1.0105*P*] where *P* = (*F*_o_^2^ + 2*F*_c_^2^)/3
3796 reflections	(Δ/σ)_max_ < 0.001
259 parameters	Δρ_max_ = 0.21 e Å^−^^3^
0 restraints	Δρ_min_ = −0.28 e Å^−^^3^

### Special details

**Table:**

Geometry. Bond distances, angles *etc*. have been calculated using the rounded fractional coordinates. All su's are estimated from the variances of the (full) variance-covariance matrix. The cell e.s.d.'s are taken into account in the estimation of distances, angles and torsion angles
Refinement. Refinement of *F*^2^ against ALL reflections. The weighted *R*-factor *wR* and goodness of fit *S* are based on *F*^2^, conventional *R*-factors *R* are based on *F*, with *F* set to zero for negative *F*^2^. The threshold expression of *F*^2^ > σ(*F*^2^) is used only for calculating *R*-factors(gt) *etc*. and is not relevant to the choice of reflections for refinement. *R*-factors based on *F*^2^ are statistically about twice as large as those based on *F*, and *R*- factors based on ALL data will be even larger.

### Fractional atomic coordinates and isotropic or equivalent isotropic displacement parameters (Å^2^)

**Table:**

	*x*	*y*	*z*	*U*_iso_*/*U*_eq_	
S1	0.33210 (4)	0.16234 (8)	0.39452 (7)	0.0639 (3)	
O1	0.14274 (12)	0.0303 (2)	−0.1123 (2)	0.0874 (11)	
O2	0.07789 (15)	−0.0466 (3)	−0.2721 (3)	0.1110 (16)	
O3	0.30024 (11)	0.2317 (2)	0.46012 (18)	0.0765 (10)	
O4	0.36264 (12)	0.0762 (2)	0.43976 (18)	0.0799 (10)	
N1	0.19699 (14)	−0.0123 (3)	0.0534 (3)	0.0683 (14)	
N2	0.37120 (13)	0.2493 (3)	0.3342 (2)	0.0719 (11)	
N3	0.42863 (12)	0.3128 (3)	0.2071 (2)	0.0638 (11)	
N4	0.41470 (12)	0.1191 (3)	0.2320 (2)	0.0650 (12)	
C1	0.15104 (16)	−0.1524 (3)	−0.0429 (3)	0.0673 (17)	
C2	0.13077 (16)	−0.0756 (3)	−0.1170 (3)	0.0683 (16)	
C3	0.09721 (18)	−0.1143 (3)	−0.1978 (3)	0.0783 (17)	
C4	0.08434 (18)	−0.2239 (4)	−0.2017 (4)	0.091 (2)	
C5	0.10447 (18)	−0.2985 (3)	−0.1295 (4)	0.0843 (19)	
C6	0.13772 (17)	−0.2643 (3)	−0.0517 (3)	0.0773 (17)	
C7	0.18412 (16)	−0.1152 (3)	0.0396 (3)	0.0700 (17)	
C8	0.22986 (15)	0.0266 (3)	0.1351 (3)	0.0567 (14)	
C9	0.27177 (16)	−0.0346 (3)	0.1735 (3)	0.0667 (16)	
C10	0.30228 (15)	0.0054 (3)	0.2545 (3)	0.0653 (14)	
C11	0.29108 (14)	0.1070 (3)	0.2961 (2)	0.0523 (12)	
C12	0.24920 (16)	0.1698 (3)	0.2564 (3)	0.0633 (14)	
C13	0.21849 (16)	0.1290 (3)	0.1757 (3)	0.0653 (14)	
C14	0.40682 (15)	0.2243 (3)	0.2534 (3)	0.0613 (16)	
C15	0.46458 (16)	0.2897 (3)	0.1314 (3)	0.0663 (16)	
C16	0.47691 (16)	0.1837 (3)	0.1050 (3)	0.0730 (16)	
C17	0.45058 (16)	0.0989 (3)	0.1559 (3)	0.0683 (16)	
C18	0.45913 (17)	−0.0204 (3)	0.1284 (3)	0.0923 (19)	
C19	0.48948 (18)	0.3859 (3)	0.0768 (3)	0.0920 (19)	
H1	0.1840 (16)	0.024 (3)	0.010 (3)	0.0821*	
H2	0.08717	0.01670	−0.25928	0.1666*	
H2A	0.36975	0.31672	0.35398	0.0861*	
H4	0.06153	−0.24894	−0.25413	0.1089*	
H5	0.09512	−0.37230	−0.13437	0.1011*	
H6	0.15163	−0.31479	−0.00446	0.0928*	
H7	0.19743	−0.16678	0.08640	0.0841*	
H9	0.27959	−0.10292	0.14484	0.0801*	
H10	0.33052	−0.03633	0.28130	0.0782*	
H12	0.24188	0.23889	0.28391	0.0759*	
H13	0.19016	0.17036	0.14875	0.0779*	
H16	0.50251	0.16893	0.05389	0.0876*	
H18A	0.43576	−0.04011	0.07173	0.1383*	
H18B	0.45100	−0.06537	0.18778	0.1383*	
H18C	0.49606	−0.03159	0.10819	0.1383*	
H19A	0.46303	0.42001	0.03270	0.1379*	
H19B	0.51940	0.36133	0.03535	0.1379*	
H19C	0.50198	0.43815	0.12743	0.1379*	

### Atomic displacement parameters (Å^2^)

**Table:**

	*U*^11^	*U*^22^	*U*^33^	*U*^12^	*U*^13^	*U*^23^
S1	0.0759 (7)	0.0645 (6)	0.0512 (5)	−0.0047 (6)	0.0066 (5)	−0.0030 (5)
O1	0.112 (2)	0.0531 (16)	0.097 (2)	0.0023 (16)	−0.0265 (17)	−0.0057 (15)
O2	0.141 (3)	0.080 (2)	0.112 (3)	0.012 (2)	−0.051 (2)	−0.016 (2)
O3	0.090 (2)	0.0805 (18)	0.0591 (15)	−0.0076 (15)	0.0199 (14)	−0.0167 (14)
O4	0.099 (2)	0.0790 (18)	0.0618 (16)	0.0104 (16)	−0.0146 (15)	0.0079 (14)
N1	0.083 (3)	0.060 (2)	0.062 (2)	0.0034 (19)	−0.0070 (19)	0.0035 (18)
N2	0.081 (2)	0.0598 (18)	0.075 (2)	−0.0124 (17)	0.0241 (19)	−0.0180 (17)
N3	0.066 (2)	0.068 (2)	0.0573 (19)	−0.0040 (17)	−0.0016 (16)	0.0028 (16)
N4	0.065 (2)	0.068 (2)	0.062 (2)	−0.0039 (17)	0.0091 (17)	−0.0125 (17)
C1	0.066 (3)	0.062 (3)	0.074 (3)	−0.001 (2)	0.005 (2)	−0.007 (2)
C2	0.071 (3)	0.057 (2)	0.077 (3)	−0.002 (2)	−0.001 (2)	−0.019 (2)
C3	0.079 (3)	0.070 (3)	0.086 (3)	0.003 (2)	−0.010 (3)	−0.015 (3)
C4	0.090 (4)	0.073 (3)	0.109 (4)	−0.009 (3)	−0.010 (3)	−0.029 (3)
C5	0.084 (3)	0.059 (3)	0.110 (4)	−0.011 (2)	0.009 (3)	−0.017 (3)
C6	0.080 (3)	0.059 (3)	0.093 (3)	−0.006 (2)	0.016 (3)	0.000 (2)
C7	0.071 (3)	0.062 (3)	0.077 (3)	0.003 (2)	0.009 (2)	0.005 (2)
C8	0.068 (3)	0.051 (2)	0.051 (2)	−0.003 (2)	−0.0007 (19)	0.0017 (19)
C9	0.076 (3)	0.051 (2)	0.073 (3)	0.009 (2)	−0.002 (2)	−0.004 (2)
C10	0.072 (3)	0.055 (2)	0.069 (2)	0.008 (2)	−0.007 (2)	−0.002 (2)
C11	0.061 (2)	0.047 (2)	0.049 (2)	−0.0016 (18)	0.0038 (17)	0.0042 (17)
C12	0.086 (3)	0.048 (2)	0.056 (2)	0.006 (2)	0.013 (2)	−0.002 (2)
C13	0.076 (3)	0.060 (2)	0.060 (2)	0.015 (2)	−0.001 (2)	0.005 (2)
C14	0.061 (3)	0.066 (3)	0.057 (2)	−0.008 (2)	0.002 (2)	−0.008 (2)
C15	0.061 (3)	0.087 (3)	0.051 (2)	−0.006 (2)	−0.004 (2)	0.006 (2)
C16	0.071 (3)	0.091 (3)	0.057 (2)	0.001 (2)	0.009 (2)	−0.004 (2)
C17	0.068 (3)	0.081 (3)	0.056 (2)	0.002 (2)	−0.002 (2)	−0.004 (2)
C18	0.106 (4)	0.088 (3)	0.083 (3)	0.011 (3)	0.017 (3)	−0.018 (3)
C19	0.095 (4)	0.104 (3)	0.077 (3)	−0.013 (3)	0.018 (2)	0.023 (3)

### Geometric parameters (Å, º)

**Table:**

S1—O3	1.430 (3)	C8—C13	1.380 (5)
S1—O4	1.417 (3)	C9—C10	1.374 (5)
S1—N2	1.630 (3)	C10—C11	1.375 (5)
S1—C11	1.755 (3)	C11—C12	1.385 (5)
O1—C2	1.324 (4)	C12—C13	1.378 (5)
O2—C3	1.348 (5)	C15—C16	1.368 (5)
O2—H2	0.8200	C15—C19	1.497 (5)
N1—C7	1.304 (5)	C16—C17	1.385 (5)
N1—C8	1.409 (5)	C17—C18	1.509 (5)
N2—C14	1.395 (5)	C4—H4	0.9300
N3—C15	1.347 (5)	C5—H5	0.9300
N3—C14	1.343 (5)	C6—H6	0.9300
N4—C14	1.324 (5)	C7—H7	0.9300
N4—C17	1.343 (5)	C9—H9	0.9300
N1—H1	0.78 (4)	C10—H10	0.9300
N2—H2A	0.8600	C12—H12	0.9300
C1—C2	1.425 (5)	C13—H13	0.9300
C1—C6	1.406 (5)	C16—H16	0.9300
C1—C7	1.413 (5)	C18—H18A	0.9600
C2—C3	1.410 (6)	C18—H18B	0.9600
C3—C4	1.372 (6)	C18—H18C	0.9600
C4—C5	1.390 (7)	C19—H19A	0.9600
C5—C6	1.360 (6)	C19—H19B	0.9600
C8—C9	1.369 (5)	C19—H19C	0.9600
			
O3—S1—O4	119.31 (15)	N3—C14—N4	128.6 (3)
O3—S1—N2	102.96 (16)	N2—C14—N3	114.1 (3)
O3—S1—C11	109.37 (16)	N3—C15—C19	116.5 (3)
O4—S1—N2	111.00 (17)	C16—C15—C19	122.0 (4)
O4—S1—C11	108.66 (17)	N3—C15—C16	121.5 (4)
N2—S1—C11	104.50 (15)	C15—C16—C17	118.7 (4)
C3—O2—H2	109.00	N4—C17—C18	116.0 (3)
C7—N1—C8	124.4 (4)	C16—C17—C18	122.7 (3)
S1—N2—C14	126.0 (3)	N4—C17—C16	121.2 (3)
C14—N3—C15	114.7 (3)	C3—C4—H4	119.00
C14—N4—C17	115.2 (3)	C5—C4—H4	119.00
C7—N1—H1	110 (3)	C4—C5—H5	120.00
C8—N1—H1	125 (3)	C6—C5—H5	120.00
C14—N2—H2A	117.00	C1—C6—H6	120.00
S1—N2—H2A	117.00	C5—C6—H6	120.00
C2—C1—C7	119.6 (3)	N1—C7—H7	118.00
C2—C1—C6	119.9 (4)	C1—C7—H7	118.00
C6—C1—C7	120.4 (3)	C8—C9—H9	120.00
C1—C2—C3	118.7 (3)	C10—C9—H9	120.00
O1—C2—C1	122.0 (3)	C9—C10—H10	120.00
O1—C2—C3	119.4 (3)	C11—C10—H10	120.00
C2—C3—C4	119.2 (4)	C11—C12—H12	120.00
O2—C3—C2	121.7 (3)	C13—C12—H12	120.00
O2—C3—C4	119.1 (4)	C8—C13—H13	120.00
C3—C4—C5	121.8 (4)	C12—C13—H13	120.00
C4—C5—C6	120.5 (4)	C15—C16—H16	121.00
C1—C6—C5	119.8 (4)	C17—C16—H16	121.00
N1—C7—C1	123.4 (4)	C17—C18—H18A	109.00
N1—C8—C9	121.5 (3)	C17—C18—H18B	109.00
C9—C8—C13	120.7 (4)	C17—C18—H18C	109.00
N1—C8—C13	117.8 (3)	H18A—C18—H18B	109.00
C8—C9—C10	119.8 (3)	H18A—C18—H18C	109.00
C9—C10—C11	120.1 (3)	H18B—C18—H18C	109.00
S1—C11—C10	120.5 (3)	C15—C19—H19A	109.00
S1—C11—C12	119.1 (3)	C15—C19—H19B	109.00
C10—C11—C12	120.3 (3)	C15—C19—H19C	109.00
C11—C12—C13	119.4 (3)	H19A—C19—H19B	109.00
C8—C13—C12	119.8 (4)	H19A—C19—H19C	109.00
N2—C14—N4	117.3 (3)	H19B—C19—H19C	109.00
			
O3—S1—N2—C14	−173.5 (3)	C2—C1—C6—C5	−1.6 (6)
O4—S1—N2—C14	57.7 (3)	C7—C1—C6—C5	177.9 (4)
C11—S1—N2—C14	−59.3 (3)	C2—C1—C7—N1	1.0 (6)
O3—S1—C11—C10	−149.8 (3)	C6—C1—C7—N1	−178.5 (4)
O3—S1—C11—C12	34.1 (3)	O1—C2—C3—O2	1.6 (6)
O4—S1—C11—C10	−18.0 (3)	O1—C2—C3—C4	−179.2 (4)
O4—S1—C11—C12	165.9 (3)	C1—C2—C3—O2	−178.1 (4)
N2—S1—C11—C10	100.5 (3)	C1—C2—C3—C4	1.1 (6)
N2—S1—C11—C12	−75.6 (3)	O2—C3—C4—C5	177.8 (4)
C8—N1—C7—C1	180.0 (4)	C2—C3—C4—C5	−1.5 (7)
C7—N1—C8—C9	34.0 (6)	C3—C4—C5—C6	0.3 (7)
C7—N1—C8—C13	−146.0 (4)	C4—C5—C6—C1	1.2 (7)
S1—N2—C14—N3	170.9 (3)	N1—C8—C9—C10	−178.8 (4)
S1—N2—C14—N4	−8.9 (5)	C13—C8—C9—C10	1.1 (6)
C15—N3—C14—N2	177.7 (3)	N1—C8—C13—C12	179.3 (4)
C15—N3—C14—N4	−2.5 (6)	C9—C8—C13—C12	−0.7 (6)
C14—N3—C15—C16	0.3 (5)	C8—C9—C10—C11	−0.7 (6)
C14—N3—C15—C19	179.4 (3)	C9—C10—C11—S1	−176.4 (3)
C17—N4—C14—N2	−178.0 (3)	C9—C10—C11—C12	−0.3 (5)
C17—N4—C14—N3	2.2 (6)	S1—C11—C12—C13	176.9 (3)
C14—N4—C17—C16	0.1 (5)	C10—C11—C12—C13	0.8 (5)
C14—N4—C17—C18	−178.5 (3)	C11—C12—C13—C8	−0.3 (6)
C6—C1—C2—O1	−179.3 (4)	N3—C15—C16—C17	1.7 (6)
C6—C1—C2—C3	0.4 (6)	C19—C15—C16—C17	−177.3 (4)
C7—C1—C2—O1	1.2 (6)	C15—C16—C17—N4	−1.9 (6)
C7—C1—C2—C3	−179.1 (4)	C15—C16—C17—C18	176.6 (4)

### Hydrogen-bond geometry (Å, º)

**Table:**

*D*—H···*A*	*D*—H	H···*A*	*D*···*A*	*D*—H···*A*
N1—H1···O1	0.78 (4)	1.88 (4)	2.569 (5)	148 (4)
O2—H2···O1	0.82	2.34	2.768 (5)	113
O2—H2···N3^i^	0.82	2.16	2.862 (5)	144
N2—H2*A*···O1^ii^	0.86	1.94	2.790 (4)	172
C18—H18*A*···O4^iii^	0.96	2.52	3.469 (5)	171

Symmetry codes: (i) −*x*+1/2, −*y*+1/2, *z*−1/2; (ii) −*x*+1/2, −*y*+1/2, *z*+1/2; (iii) *x*, −*y*, *z*−1/2.
